# Impact of maintaining mild mitral regurgitation beyond 1 year after mitral transcatheter edge-to-edge repair

**DOI:** 10.1093/eschf/xvag016

**Published:** 2026-01-14

**Authors:** Azusa Kurita, Masanori Yamamoto, Tetsuro Shimura, Ai Kagase, Takahiro Tokuda, Atsushi Sugiura, Hiroshi Tsunamoto, Ryo Yamaguchi, Mike Saji, Yuki Izumi, Masahiko Asami, Yusuke Enta, Daisuke Hachinohe, Shinichi Shirai, Masaki Izumo, Shingo Mizuno, Yusuke Watanabe, Makoto Amaki, Kazuhisa Kodama, Hisao Otsuki, Toru Naganuma, Hiroki Bota, Yohei Ohno, Masahiro Yamawaki, Hiroshi Ueno, Gaku Nakazawa, Toshiaki Otsuka, Shunsuke Kubo, Kentaro Hayashida

**Affiliations:** Department of Cardiology, Gifu Heart Center, Gifu, Japan; Department of Cardiology, Gifu Heart Center, Gifu, Japan; Department of Cardiology, Nagoya Heart Center, Nagoya, Japan; Department of Cardiology, Toyohashi Heart Center, Toyohashi, Japan; Department of Cardiology, Gifu Heart Center, Gifu, Japan; Department of Cardiology, Nagoya Heart Center, Nagoya, Japan; Department of Cardiology, Nagoya Heart Center, Nagoya, Japan; Department of Cardiology, Nagoya Heart Center, Nagoya, Japan; Department of Cardiology, Nagoya Heart Center, Nagoya, Japan; Department of Cardiology, Toyohashi Heart Center, Toyohashi, Japan; Department of Cardiology, Sakakibara Heart Institute, Tokyo, Japan; Division of Cardiovascular Medicine, Department of Internal Medicine, Toho University Faculty of Medicine, Tokyo, Japan; Department of Cardiology, Sakakibara Heart Institute, Tokyo, Japan; Division of Cardiology, Mitsui Memorial Hospital, Tokyo, Japan; Department of Cardiology, Sendai Kosei Hospital, Sendai, Japan; Division of Cardiology, Sapporo Heart Center, Sapporo, Japan; Department of Cardiology, Kokura Memorial Hospital, Kitakyushu, Japan; Division of Cardiology, St. Marianna University School of Medicine Hospital, Kawasaki, Japan; Department of Cardiology, Shonan Kamakura General Hospital, Kanagawa, Japan; Department of Cardiology, Teikyo University School of Medicine, Tokyo, Japan; Department of Heart Failure and Transplant Division of Heart Failure, National Cerebral and Cardiovascular Center, Suita, Japan; Division of Cardiology, Saiseikai Kumamoto Hospital Cardiovascular Center, Kumamoto, Japan; Department of Cardiology, Tokyo Woman’s Medical University, Tokyo, Japan; Department of Cardiology, New Tokyo Hospital, Chiba, Japan; Department of Cardiology, Sapporo Higashi Tokushukai Hospital, Sapporo, Japan; Department of Cardiology, Tokai University School of Medicine, Isehara, Japan; Department of Cardiology, Saiseikai Yokohama City Eastern Hospital, Kanagawa, Japan; Second Department of Internal Medicine, Toyama University Hospital, Toyama, Japan; Division of Cardiology, Department of Medicine, Kindai University Faculty of Medicine, Osaka, Japan; Department of Hygiene and Public Health, Nippon Medical School, Tokyo, Japan; Center for Clinical Research, Nippon Medical School Hospital, Tokyo, Japan; Department of Cardiology, Kurashiki Central Hospital, Kurashiki, Japan; Department of Cardiology, Keio University School of Medicine, Tokyo, Japan

**Keywords:** Mitral transcatheter edge-to-edge repair, Stable mitral regurgitation, Worsening mitral regurgitation, Long-term outcome

## Abstract

**Introduction:**

Residual mitral regurgitation (MR) after mitral transcatheter edge-to-edge repair (M-TEER) is associated with adverse prognosis. However, the long-term clinical impact of MR persistence or progression has not been well stratified. We aimed to evaluate the proportion, clinical benefits, and prognostic value of maintaining mild MR 1 year after M-TEER.

**Methods and Results:**

This multi-centre registry-based analysis included 1865 patients who achieved mild MR at discharge following M-TEER. At 1 year, patients were classified as having stable MR (≤ mild) or worsening MR (≥ moderate). The frequency of left atrial (LA) and left ventricular (LV) reverse remodelling and tricuspid regurgitation (TR) improvement were assessed from baseline to 1 year. Clinical endpoints—including all-cause mortality and heart failure hospitalization—were evaluated beyond 1 year after M-TEER up to 2 years. Worsening MR occurred in 28.4% of patients. Compared with the worsening MR group, the stable MR group demonstrated significantly more frequent LA and LV reverse remodelling (38.4% vs 28.1%, and 44.7% vs 31.1%, respectively; both *P* < .001). Improvement in TR (≥ Grade 1) was also more prevalent in the stable MR group (32.4% vs 20.6%, *P* < .001). Worsening MR was independently associated with increased risk of adverse clinical outcomes (hazard ratio: 2.02; 95% confidence interval: 1.26–3.23; *P* = .003).

**Conclusion:**

Maintaining MR within mild at 1-year post-M-TEER is associated with favourable cardiac reverse remodelling and improved clinical prognosis. These findings underscore the importance of long-term MR surveillance and its implications for outcome optimization following M-TEER.

## Central illustration

**Central illustration:**
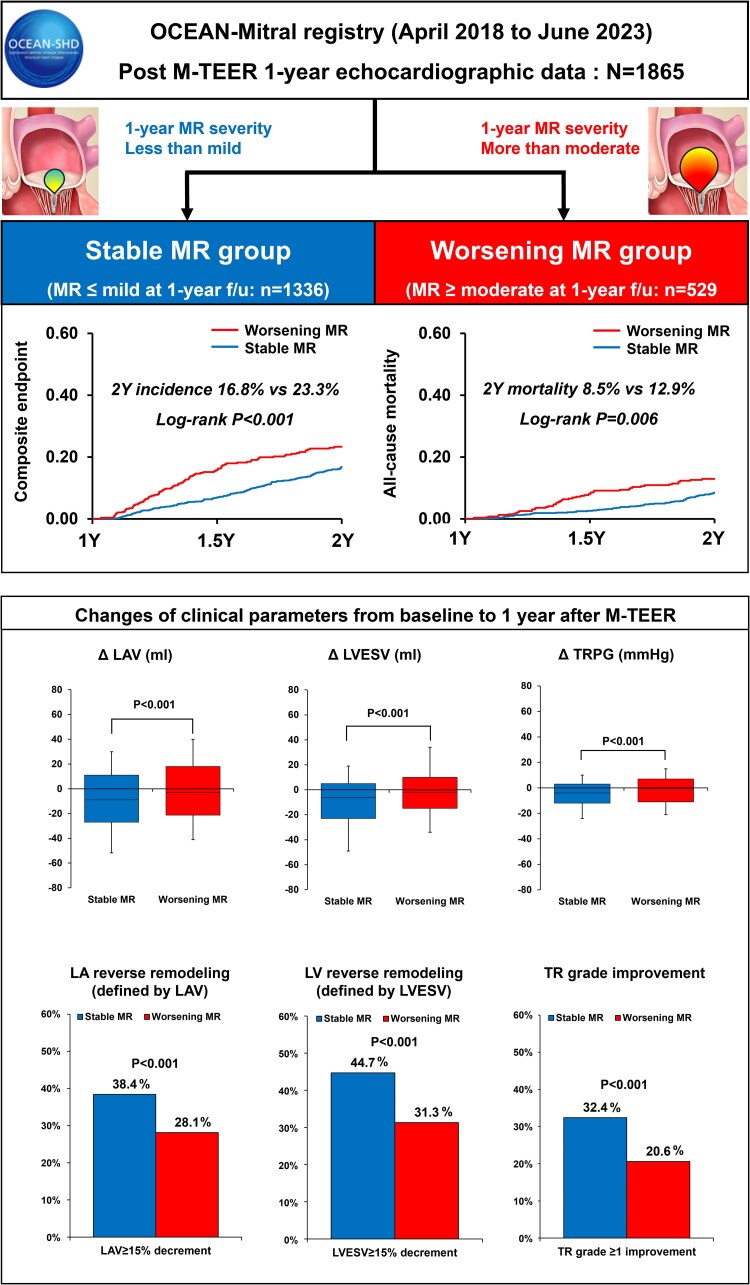
This study compared two groups of patients with mild MR immediately after M-TEER: the stable MR group (*n* = 1336), which maintained mild MR at 1 year, and the worsening MR group (*n* = 529), in which MR progressed to moderate or greater. The stable MR group had significantly lower risks of the primary outcome (all-cause death and HF hospitalization) and all-cause mortality. Moreover, from baseline to 1 year, the stable MR group showed greater improvements in cardiac structure and function, including significant reductions in LAV, LVEF, LVESV, and TRPG. Stable MR was also associated with improvement in LA and LV reverse remodelling, as well as TR grade, compared to the worsening MR group. MR, mitral regurgitation; M-TEER, mitral transcatheter edge-to-edge repair; HF, heart failure; LAV, left atrial volume; LVEF, left ventricle ejection fraction; LVESV, left ventricle end-systolic volume; TRPG, tricuspid regurgitation pressure gradient; TR, tricuspid regurgitation.

## Introduction

Mitral transcatheter edge-to-edge repair (M-TEER) has been developed as a new therapeutic approach for mitral regurgitation (MR). The previous pivotal randomized trials reveal the effectiveness of M-TEER using MitraClip (Abbott Vascular, Menlo Park, CA, USA) in patients suffering both degenerative MR (DMR) and functional MR (FMR).^1,2^ With the widespread indication of M-TEER in daily practice, careful patient screening and risk assessment should be required before invasive therapy. In addition, a detailed study of what is needed to improve patient prognosis is required.

It has been reported that even residual MR ≥ moderate after M-TEER at discharge is associated with worse clinical outcomes compared with residual MR ≤ mild.^3–5^ However, this is limited to residual MR in the early post-operative period. Although residual MR is considered important to maintain below mild, it is well established that residual MR changes dynamically during the follow-up period and tends to increase more frequently over time. Worsened MR in the chronic phase may be defined by different factors in the aetiologies of FMR and DMR. To date, the proportion, predictive factors, and clinical effects of patients with increasing MR in the chronic phase have not been fully clarified. The existence of residual MR, which often increases during the chronic phase, is also expected to have the potential for poorer patient outcomes over time. The prognostic value of the dynamic change of MR in the chronic phase should be verified to visualize the future direction of our daily practice.

The aim of this study is therefore to clarify the incidence, predictive factors, and clinical course of residual MR worsening at 1 year post-operatively, as well as to stratify the FMR and DMR patients following M-TEER. In addition, the prognostic impact of the severity of MR beyond 1 year was investigated in patients who underwent M-TEER.

## Methods

### Study design and population

**Figure 1:**
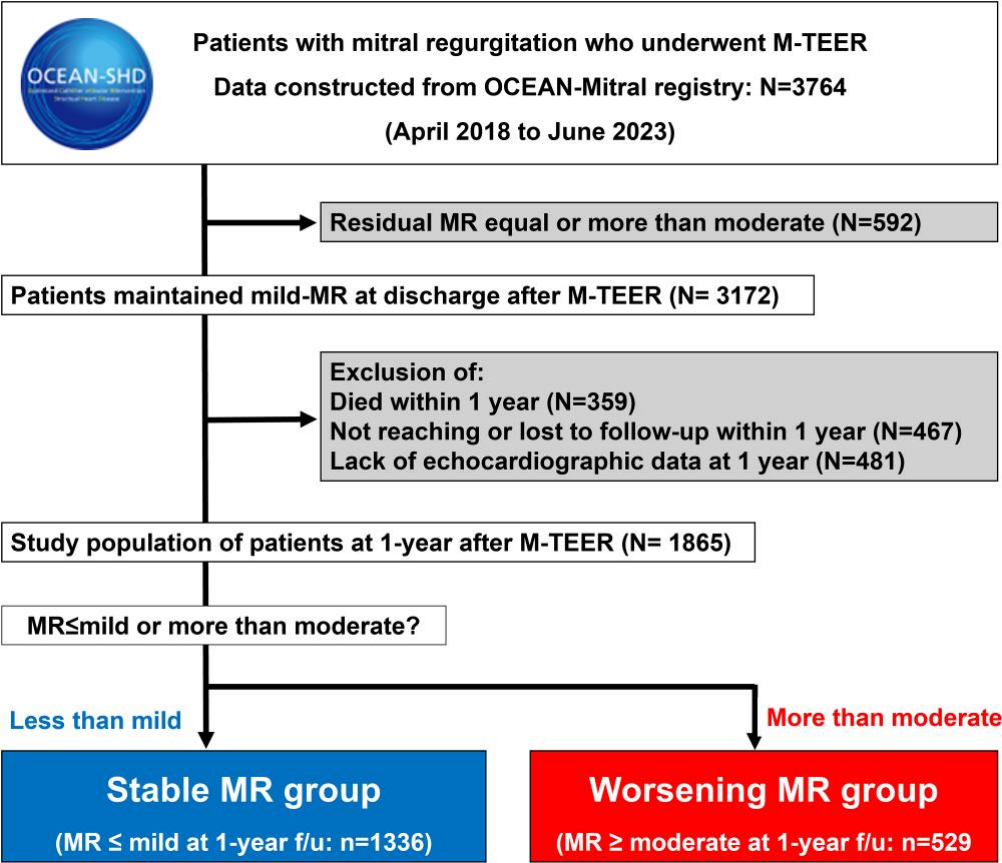
Study flow chart

The Optimized CathEter vAlvular iNtervention (OCEAN)-Mitral registry was designed to determine the safety and effectiveness of transcatheter mitral valve therapies, including M-TEER with the MitraClip for patients with symptomatic FMR and DMR.^3,6^ It is an ongoing, prospective, investigator-initiated, multi-centre, observational registry consisting of a total of 21 Japanese institutions. During the duration of the study follow-up period, a total of 3764 consecutive patients with symptomatic MR underwent TEER from April 2018 to June 2023. From April 2018 to August 2020, a second-generation (G2) system was used, and from September 2020 to June 2023, a fourth-generation (G4) system was used. The severity of MR was determined based on qualitative and quantitative criteria. The MR severity was classified as 0 (none/trivial), 1+ (mild), 2+ (moderate), 3+ (moderate to severe), or 4+ (severe). Mitral regurgitation 3+ or more at rest or exercise was considered significant MR and indicated for M-TEER. After the M-TEER procedure, MR severity was assessed according to the guideline recommendations.^7^ Transthoracic echocardiographic data at baseline, discharge, and 1 year post-operative were analysed in this study. Initially, 592 patients with more than mild MR at discharge after M-TEER were excluded from the present analysis. The MR deterioration rates before discharge were 21.7% for FMR and 28.8% for DMR. Thereafter, the 359 patients who died within 1 year, 467 patients whose observation period was <1 year, as well as those who were lost to follow-up within 1 year, and 481 patients with missing information of echocardiographic findings were excluded. This study included a final cohort of 1865 patients as the study population. Based on the echocardiographic results at 1 year after M-TEER, patients were divided into 2 groups as stable MR group (*n* = 1336) and worsening MR group (*n* = 529). The study flow chart is described in *Figure 1*. The patients were reviewed by the multidisciplinary local heart team consisting of an interventional cardiologist, a cardiothoracic surgeon, and an echocardiologist. This study was registered with the University Hospital Medical Information Network Clinical Trials Registry, as accepted by the International Committee of Medical Journal Editors (UMIN000023653). All study participants provided informed consent, and the study protocol was approved by the institutional review board of each institution. The study was conducted in accordance with the provisions of the Declaration of Helsinki.

### Clinical outcomes and definition

The detailed information regarding M-TEER was previously described.^3,6^ Baseline characteristics, laboratory data, and echocardiographic findings were assessed at each centre. Clinical follow-up was scheduled at baseline, 1 month, 1 year, and annually thereafter. The primary endpoint of this analysis was a composite of all-cause death and heart failure (HF) hospitalization from 1 to 2 years after M-TEER. The secondary endpoint was all-cause mortality. These endpoints were evaluated in the overall, FMR, and DMR cohorts, respectively. HF hospitalization was defined following the published statement: a hospitalization of at least one night due to a primary diagnosis of symptomatic HF with objective evidence of new or worsening HF, requiring initiation or intensification of treatment.^8^ The ascertainment of the primary endpoint was performed using electronic medical records and systematic telephone surveys. We also examined the influence of predisposing factors in patients with worsening residual MR at 1 year following M-TEER, distinguishing between FMR and DMR for separate analysis. At the 1-year marking comparing with baseline, the brain natriuretic peptide (BNP) values, N-terminal pro-BNP (NT-pro-BNP), New York Heart Association (NYHA) functional class, left atrium (LA) volume (LAV), left ventricle (LV) function, tricuspid regurgitation (TR), and TR pressure gradient (TRPG) were evaluated between the stable MR and worsening MR groups. For quantitative parameters among these values, the change from baseline to the 1-year mark was calculated as Δ values (1-year value minus baseline value). The echocardiographic parameter related to LA reverse remodelling is defined as a decrease in LAV ≤15% from baseline to 1 year after M-TEER. Echocardiography-related LV reverse remodelling was also defined as a decrease in LV end-systolic volume (LVESV) of ≥15%.^9,10^

### Statistical analysis

Data is presented as mean ± standard deviation or medians (first and third interquartile ranges) for continuous variables as appropriate and as frequencies (%) for categorical variables. Baseline group differences were evaluated using the χ^2^ test or Fisher’s exact for categorical covariates and Student’s *t*-test or the Mann–Whitney *U*-test for continuous variables, which depended on their distribution. Using Kaplan–Meier methods, a landmark analysis starting at 1-year post-M-TEER was performed to assess event rates of primary and secondary study endpoints up to 2 years. The log-rank tests were used for survival analysis between the stable MR and worsening MR groups. Logistic regression models were constructed to reveal predictive clinical factors of worsening MR at 1 year following M-TEER. The multivariable model adjustment included clinical variables for predicting worsening MR. In addition, predictors of MR worsening are separately analysed for atrial FMR and ventricular FMR. The Cox regression analysis was also performed to evaluate the association between the clinically significant variables and worsening MR concerning all clinical endpoints. All variables with a *P*-value <.05 in univariable analysis were incorporated into the multivariable model, alongside key clinical covariates such as age and gender. All statistical analyses were performed with SPSS software (version 25.0, SPSS). In all analyses, a two-tailed *P* < .05 indicated statistical significance.

## Results

### Baseline patient and echocardiographic characteristics

Among 1865 patients, worsening MR occurred in 529 cases (28.4%). By aetiology, DMR showed a higher 1-year incidence of worsening MR than FMR [34.5% (184/532) vs 25.9% (345/1333), *P* < .001]. The study flow chart is described in *Figure 1*, and the baseline patient characteristics are presented in *Table 1*. There were no significant differences between the stable and worsening groups, except for the body weight, body mass index (BMI), diabetes, coronary artery disease, NYHA III/IV, and beta-blocker prescription. The echocardiographic parameters and procedural variables in the 2 groups are summarized in *Table 2*. The baseline LV ejection fraction (LVEF), LAV index (LAVI), and TRPG were significantly lower in the stable group compared to the worsening MR group. The baseline MR severity of effective regurgitant orifice area, regurgitant volume, and regurgitation fraction were significantly greater in the worsening MR group than in the stable MR group.

**Table 1:** Patient characteristics

	*N*	Stable MR group (*n* = 1336)	Worsening MR group (*n* = 529)	*P-value*
Baseline clinical characteristics				
Age, years	1865	78.2 ± 9.5	78.6 ± 9.6	.354
Male, *n* (%)	1865	741 (55.5%)	279 (52.7%)	.287
Height, cm	1865	157.3 ± 10.1	156.7 ± 10.0	.205
Weight, kg	1865	53.8 ± 11.8	52.4 ± 11.2	.020
BMI, kg/m^2^	1865	21.6 ± 3.5	21.2 ± 3.4	.032
Comorbidities and general status				
Systolic BP, mmHg	1865	111.8 ± 19.0	110.5 ± 17.9	.155
Diastolic BP, mmHg	1865	64.9 ± 12.9	64.6 ± 12.2	.683
Heart rate, /min	1865	73 ± 15	74 ± 14	.302
Hypertension, *n* (%)	1865	899 (67.3%)	348 (65.8%)	.533
Dyslipidaemia, *n* (%)	1865	680 (50.9%)	262 (49.5%)	.594
Diabetes mellitus, *n* (%)	1865	370 (27.7%)	113 (21.4%)	.005
Smoking, *n* (%)	1865	453 (33.9%)	174 (32.9%)	.640
Chronic kidney disease, *n* (%)	1865	1136 (85.0%)	457 (86.4%)	.453
Dialysis, *n* (%)	1865	73 (5.5%)	23 (4.3%)	.325
COPD, *n* (%)	1865	100 (7.5%)	54 (10.2%)	.054
Coronary artery disease, *n* (%)	1865	487 (36.5%)	167 (31.6%)	.046
Atrial fibrillation, *n* (%)	1865	788 (59.0%)	327 (61.8%)	.272
NYHA Class III or IV, *n* (%)	1865	816 (61.0%)	292 (55.2%)	.020
Clinical frailty scale ≥4, *n* (%)	1787	641 (49.6%)	239 (48.3%)	.615
Prior HF admission, *n* (%)	1812	1.44 ± 1.1	1.44 ± 1.1	.990
Prior HF within 1 year, *n* (%)	1861	1.03 ± 1.0	1.00 ± 0.9	.488
EuroSCORE II, %	1599	6.5 ± 5.8	6.3 ± 6.0	.535
STS score for MV repair, %	1414	6.0 (3.4–9.6)	5.6 (3.3–9.2)	.322
STS score for MV replacement, %	1751	8.5 (5.3–12.7)	8.6 (5.6–13.3)	.637
Laboratory data				
BNP, pg/ml	1354	319.2 (160.7–638.4)	290.3 (155.8–593.3)	.292
NT-pro-BNP, pg/ml	1003	2292 (1050–4585)	2192 (821–4386)	.235
Haemoglobin, g/dl	1865	11.9 ± 1.9	11.8 ± 1.7	.411
Albumin, g/dl	1857	3.7 ± 0.5	3.47 ± 0.5	.441
Creatinine, mg/dl	1856	1.6 ± 1.4	1.6 ± 1.5	.682
eGFR, mL/min/1.73m^2^	1856	40.3 ± 19.8	40.8 ± 18.4	.598
Sodium, mEq/L	1863	139.3 ± 3.3	139.3 ± 3.5	.989
Medication				
Loop diuretics, *n* (%)	1865	1062 (79.5%)	426 (80.5%)	.654
Tolvaptan, *n* (%)	1865	532 (39.8%)	207 (39.1%)	.791
ARNI, *n* (%)	1864	113 (8.5%)	43 (8.1%)	.853
Beta-blocker, *n* (%)	1833	1021 (77.8%)	373 (71.7%)	.008
MRA, *n* (%)	1862	738 (55.3%)	282 (53.5%)	.502
SGLT-2 inhibitor, *n* (%)	1865	229 (17.1%)	81 (15.3%)	.370

Values are numbers (%) or mean ± standard deviation.

ARNI, angiotensin receptor neprilysin inhibitor; BMI, body mass index; BNP, B-type natriuretic peptide; BP, blood pressure; COPD, chronic obstructive pulmonary disease; eGFR, estimated glomerular filtration ratio; HF, heart failure; MRA, mineralocorticoid receptor antagonist; MV, mitral valve; NYHA, New York Heart Association; SGLT-2, sodium glucose co-transporter-2; STS score, Society of Thoracic Surgeons score.

**Table 2:** Echocardiographic parameters and procedural variables of study patients

	*N*	Stable MR group (*n* = 1336)	Worsening MR group (*n* = 529)	*P-*value
Echo parameters before procedure				
LVEF, %	1865	44.3 ± 16.1	46.2 ± 17.1	.023
LVDd, mm	1865	56.4 ± 9.9	57.3 ± 10.6	.089
LVDs, mm	1865	43.8 ± 13.0	44.0 ± 14.0	.843
LVEDV, ml	1790	143.8 ± 66.7	149.8 ± 69.6	.091
LVESV, ml	1733	89.0 ± 58.9	92.2 ± 64.2	.325
LAVI, ml/m^2^	1811	46.3 ± 1.3	47.8 ± 2.1	<.001
TRPG, mmHg	1815	33.3 ± 13.5	34.9 ± 14.8	.027
TAPSE, mm	1528	16.5 ± 4.8	16.7 ± 4.6	.532
Mitral E wave, cm/s	1801	98.7 ± 51.0	102.9 ± 34.6	.090
e’ (septal), cm/s	1699	5.30 ± 2.4	5.67 ± 2.2	.004
E/e’ (septal), cm/s	1294	17.5 ± 8.6	16.6 ± 6.5	.060
MR EROA, cm^2^	1675	0.35 ± 0.2	0.41 ± 0.2	<.001
MR regurgitant volume, ml	1734	52.7 ± 24.3	59.0 ± 28.0	<.001
MR regurgitant fraction, %	1287	48.4 ± 18.4	52.0 ± 16.9	.002
MV orifice area, cm^2^	1492	5.2 ± 1.5	5.3 ± 1.7	.195
MV mean PG, mmHg	1521	1.8 ± 1.1	1.9 ± 1.1	.100
AS ≥moderate, *n* (%)	1780	42 (3.3%)	12 (2.4%)	.359
AR ≥moderate, *n* (%)	1861	123 (9.2%)	52 (9.8%)	.725
TR ≥moderate, *n* (%)	1861	421 (31.6%)	195 (36.9%)	.033
Pathogenesis of MR				
FMR	1333	988 (74.0%)	345 (65.2%)	<.001
DMR	532	348 (26.0%)	184 (34.8%)	
Pathogenesis of DMR	532			
Bileaflet prolapse, *n* (%)	532	61 (17.5%)	38 (20.7%)	.413
Chordal rupture, *n* (%)	532	148 (42.5%)	89 (48.4%)	.201
Central regurgitation, *n* (%)	532	136 (39.1%)	62 (33.7%)	.258
Valve flail, *n* (%)	427	80 (28.8%)	45 (30.2%)	.824
Jet direction	392			
Central	114	74 (28.2%)	40 (30.8%)	.874
Anterior	150	104 (39.7%)	46 (35.4%)	
Posterior	99	65 (24.8%)	34 (26.2%)	
Others	29	19 (7.3%)	10 (7.7%)	
Pathogenesis of FMR	1333			
Atrial functional	300	224 (16.8%)	76 (14.4%)	.209
Ventricular Functional	1033	764 (77.3%)	269 (78.0%)	.823
Coaptation gap, mm	79	2.6 ± 1.1	1.6 ± 1.4	.974
Coaptation length, mm	891	3.0 ± 2.3	2.9 ± 1.3	.383
Tenting height, mm	1036	7.8 ± 3.9	7.4 ± 3.6	.139
Procedural variables				
G2 device use, *n* (%)	1865	638 (47.8%)	256 (48.4%)	.837
Long clip if used 1 clip, *n* (%)	1332	189 (19.0)	73 (20.2)	.781
Wide clip if used 1 clip, *n* (%)	1332	475 (49.0)	162 (44.8)	.170
Number of clip, *n*	1865	1.28 ± 0.5	1.33 ± 0.5	.056
Post MV mean PG, mmHg	1744	2.9 ± 1.9	2.9 ± 1.4	.795

Values are numbers (%) or mean ± SD.

AR, aortic regurgitation; AS, aortic stenosis; DMR, degenerative mitral regurgitation; EROA, effective regurgitant orifice area; FMR, functional mitral regurgitation; G2, generation 2; LAVI, left atrial volume index; LVDd, left ventricular end-diastolic diameter; LVDs, left ventricular end-systolic diameter; LVEDV, left ventricular end-diastolic volume; LVEF, left ventricular ejection fraction; LVESV, left ventricular end-systolic volume; MR, mitral regurgitation; MV, mitral valve; PG, pressure gradient; TAPSE, tricuspid annular plane systolic excursion; TR, tricuspid regurgitation; TRPG, tricuspid regurgitation pressure gradient.

### Clinical outcomes on worsening MR stratified at 1 year after M-TEER during the chronic phase

**Figure 2:**
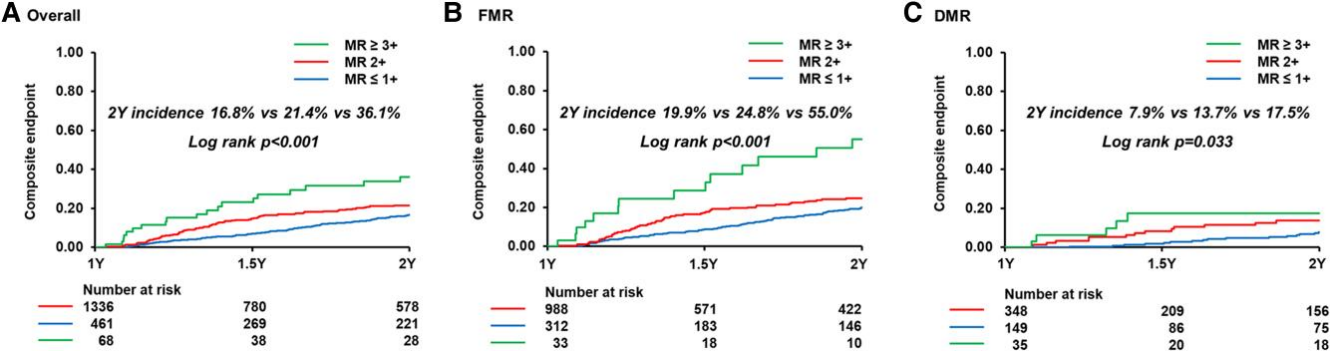
The cumulative incidence of the composite endpoint among the patients with mitral regurgitation ≤1+ (none-mild), mitral regurgitation 2+ (moderate), and mitral regurgitation ≥3+ (moderate-to-severe and severe). The incidence of composite endpoint including all-cause death and heart failure hospitalization in the overall cohort (*A*), patients with functional mitral regurgitation (*B*), and patients with degenerative mitral regurgitation (*C*)

**Figure 3:**
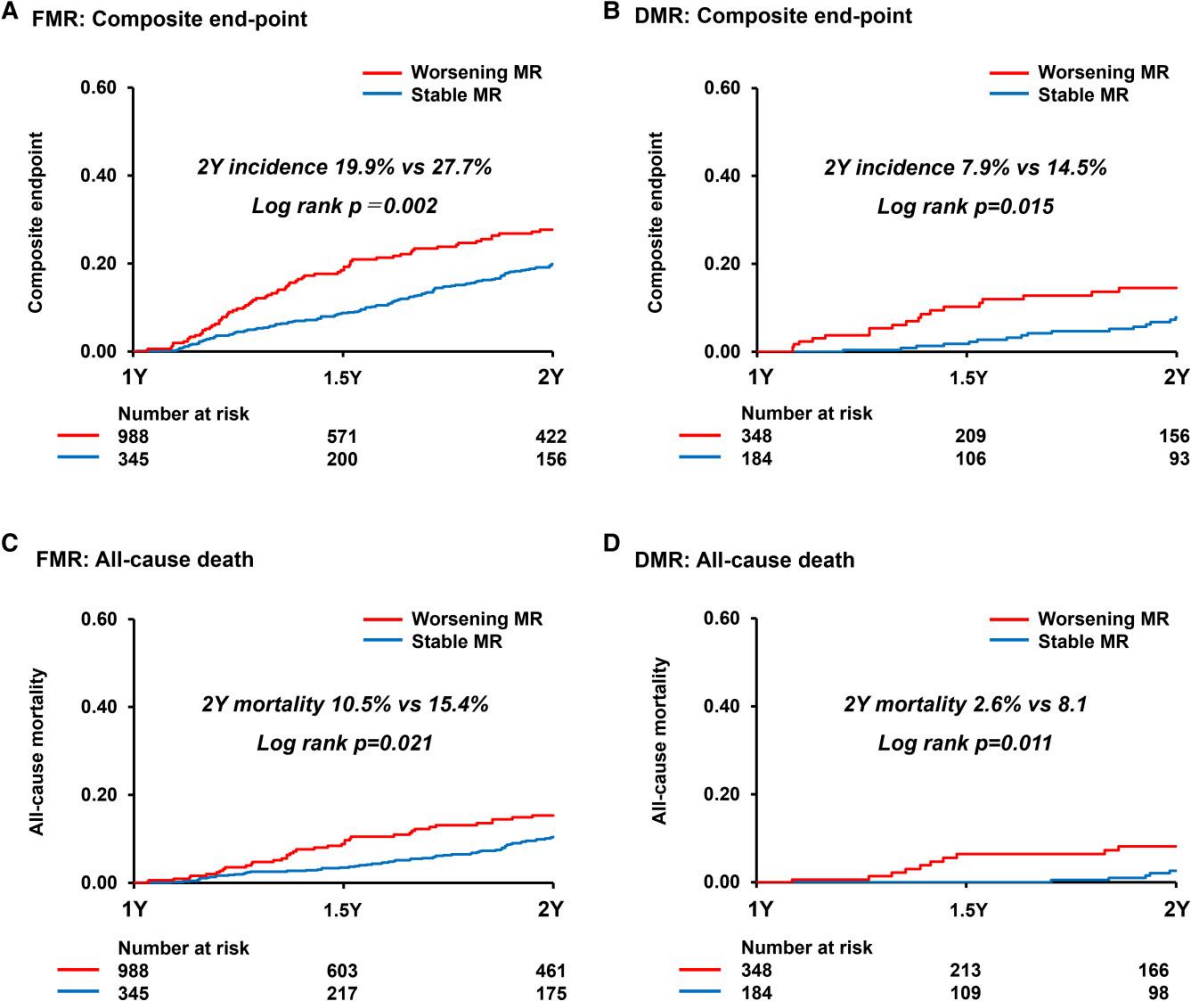
The cumulative incidence of the composite endpoint and all-cause death between the stable mitral regurgitation and worsening mitral regurgitation. The incidence of the composite endpoint in patients with functional mitral regurgitation (*A*) and degenerative mitral regurgitation (*B*). The incidence of all-cause death in patients with functional mitral regurgitation (*C*) and degenerative mitral regurgitation (*D*)

During the median follow-up of 727 (330–2019) days, a total of 370 events (118 deaths, 252 HF hospitalizations) occurred. The landmark analysis of the primary and secondary endpoints of this study was presented in the Central Illustration. The cumulative incidence of the composite endpoint beyond 1 year was significantly higher in the worsening MR group than that of the stable MR group from 1 to 2 years post-M-TEER (16.8% vs 23.3%, *P* < .001). Significant differences in all-cause mortality were also observed between the two groups (8.5% vs 12.9%, *P* = .006). Residual MR at 1 year was categorized as MR ≤1+ (*n* = 1336), MR 2+ (*n* = 461), and MR ≥3+ (*n* = 68). From the 1-year landmark, there was a stepwise increase in the risk of the composite endpoint across these groups, with higher residual MR associated with worse outcomes (2-year incidence 16.8% vs 21.4% vs 36.1%; log-rank *P* < 0.001; *Figure 2*). This trend appeared more pronounced in FMR (2-year incidence 19.9% vs 24.8% vs 55.0%) than in DMR (7.9% vs 13.7% vs 17.5%). Classified into DMR and FMR cohort, the composite clinical outcomes and all-cause mortality were presented in *Figure 3*. The better outcomes of stable MR over worsening MR were consistently observed regardless of MR aetiology. The Cox regression multivariable analysis was presented for evaluating the clinical factors for predicting the composite endpoint (*Table 3*). The worsening MR group was associated with a higher risk of the composite endpoint, which remained significant even after adjusting for several clinical confounders [hazard ratio (HR): 1.80, 95% confidence interval (CI): 1.30–2.49, *P* < .001]. Other significant clinical variables included FMR for an increased risk of composite clinical outcomes (HR: 1.87, 95% CI: 1.13–3.10, *P* = .014). In *Table 4*, the Cox regression multivariable model also shows the association between all-cause mortality and clinical findings. The worsening MR was an independent predictive factor of all-cause mortality (HR: 1.79, 95% CI: 1.12–2.87, *P* = .015). The other significant risk factors of all-cause death were male, BMI, and FMR (all *P* < .05).

**Table 3:** Cox regression multivariable models of the association between composite clinical outcomes and clinical variables

	Univariable analysis			Multivariable analysis		
Overall cohort (*n* = 1865)	HR	95% CI	*P*-value	HR	95% CI	*P*-value
MR severity 1 year after M-TEER						
Worsening MR (for Stable MR)	1.56	1.20–2.04	.001	1.80	1.30–2.49	<.001
Clinical variables						
Age, years	1.01	0.99–1.02	.374	1.01	0.99–1.03	.605
Male	1.13	0.87–1.47	.351	1.02	0.72–1.42	.932
BMI, kg/m^2^	0.95	0.91–0.99	.009	0.99	0.94–1.04	.580
Systolic BP, mmHg	0.99	0.99–1.00	.028	0.99	0.99–1.01	.361
NYHA class III/IV	1.60	1.21–2.11	.001	1.22	0.85–1.74	.281
Clinical frailty scale ≥4	1.35	1.04–1.76	.026	1.15	0.81–1.63	.430
Euro Score II	1.04	1.02–1.06	<.001	1.01	0.98–1.03	.672
Albumin, g/dl	0.58	0.45–0.73	<.001	0.80	0.56–1.12	.192
Haemoglobin, mg/dl	0.87	0.81–0.94	<.001	0.96	0.86–1.06	.415
Sodium, mEq/L	0.96	0.93–0.99	.021	0.98	0.94–1.02	.363
eGFR, mL/min/1.73m^2^	0.99	0.98–0.99	<.001	0.99	0.99–1.00	.206
Hypertension	1.01	0.77–1.34	.921			
Diabetes mellitus	1.12	1.01–1.32	.029	1.14	0.81–1.62	.447
COPD	1.35	0.91–2.02	.139			
Dialysis dependent	1.27	0.72–2.22	.407			
Atrial fibrillation/flutter	1.08	0.93–1.24	.336			
Previous stroke	1.03	0.68–1.55	.897			
LVEF, %	0.99	0.98–0.99	<.001	0.99	0.98–1.01	.306
LAVI, ml/m^2^	1.00	1.00–1.00	.177			
MV mean PG, mmHg	1.00	1.00–1.00	.536			
TRPG, mmHg	1.00	0.99–1.01	.401			
TAPSE, mm	0.96	0.93–0.99	.012	0.98	0.95–1.02	.258
Stroke volume, ml	1.00	0.99–1.01	.607			
MR EROA, cm^2^	1.06	0.49–2.28	.886			
MR regurgitation volume, ml	1.00	0.99–1.00	.378			
MV orifice area, cm^2^	1.00	1.00–1.00	.176			
FMR	2.32	1.62–3.33	<.001	1.87	1.13–3.10	.014
G2 clip use	0.84	0.65–1.09	.197			
Long clip when used 1 clip	0.79	0.46–1.35	.393			
Wide clip when used 1 clip	1.03	0.75–1.42	.848			

Abbreviations as seen in *Tables 1* and *2*.

**Table 4:** Cox regression multivariable models of the association between all-cause mortality and clinical variables

	Univariable analysis			Multivariable analysis		
Overall cohort (*n* = 1865)	HR	95% CI	*P*-value	HR	95% CI	*P*-value
MR severity 1 year after M-TEER						
Worsening MR (for Stable MR)	1.67	1.15–2.40	.007	1.79	1.12–2.87	.015
Clinical variables						
Age, years	1.02	1.00–1.04	.108	1.02	0.99–1.05	.322
Male	1.44	0.98–2.10	.060	1.69	1.02–2.80	.044
BMI, kg/m^2^	0.90	0.85–0.95	<.001	0.91	0.84–0.99	.020
Systolic BP, mmHg	0.99	0.98–0.99	.027	0.99	0.98–1.01	.349
NYHA class III/IV	1.01	0.69–1.50	.945			
Clinical frailty scale ≥4	1.01	0.83–1.23	.921			
Euro Score II	1.78	0.90–3.51	.096			
Albumin, g/dl	1.24	1.01–1.52	.045	1.16	0.69–1.94	.581
Haemoglobin, mg/dl	2.00	1.33–3.01	.001	1.19	0.70–2.02	.522
Sodium, mEq/L	1.64	1.14–2.38	.009	1.43	0.86–2.36	.168
eGFR, mL/min/1.73m^2^	1.05	1.03–1.08	<.001	1.01	0.98–1.05	.516
Hypertension	1.00	1.00–1.00	.052			
Diabetes mellitus	1.00	1.00–1.00	.086			
COPD	0.84	0.75–0.93	.001	0.96	0.83–1.11	.568
Dialysis dependent	0.39	0.29–0.54	<.001	0.67	0.41–1.10	.114
Atrial fibrillation/flutter	0.99	0.98–1.00	.150			
Previous stroke	0.96	0.91–1.01	.117			
LVEF, %	0.98	0.97–0.99	.005	1.00	0.98–1.02	.814
LAVI, ml/m^2^	1.00	1.00–1.00	.217			
MV mean PG, mmHg	1.00	0.98–1.01	.730			
TRPG, mmHg	0.95	0.90–0.99	.012	0.97	0.92–1.02	.236
TAPSE, mm	1.00	1.00–1.00	.563			
Stroke volume, ml	1.79	0.66–4.85	.254			
MR EROA, cm^2^	1.00	0.99–1.01	.908			
MR regurgitation volume, ml	1.00	1.00–1.00	.238			
MV orifice area, cm^2^	2.77	1.61–4.76	<.001	2.85	1.32–6.14	.008
FMR	0.71	0.49–1.01	.059			
G2 clip use	0.77	0.35–1.68	.511			
Long clip when used 1 clip	1.21	0.77–1.89	.409			

Abbreviations as seen in *Tables 1* and *2*.

### Predictive factors of worsening MR at 1 year after M-TEER

Multivariable analysis reveals the independent predictors of 1-year MR worsening in *Table 5* (overall cohorts). In the DMR cohort, greater MR regurgitant volume and a larger prolapse gap were independently associated with 1-year MR worsening [regurgitant volume: odds ratio (OR) 1.017, 95% CI 1.003–1.032, *P* = .018; prolapse gap: OR 1.157 per unit, 95% CI 1.049–1.277, *P* = .003]. In the FMR cohort, larger LVEDV and LAVI were independently associated with risk of 1-year MR worsening (LVEDV: OR 1.003, 95% CI 1.001–1.005, *P* = .012; LAVI: OR 1.004 per mL/m^2^, 95% CI 1.001–1.007, *P* = .005), whereas higher stroke volume was protective (OR 0.989 per mL, 95% CI 0.980–0.998, *P* = .015). The subtype-specific models for ventricular FMR and atrial FMR are presented in Supplementary Table S1, in which LVEDV and LAVI remained independent predictors in ventricular FMR. In contrast, only stroke volume showed an association in atrial FMR.

**Table 5:** Predictive risk factors of worsening mitral regurgitation at 1 year following mitral transcatheter edge-to-edge repair

Variables	Univariable analysis			Multivariable analysis		
	OR	95% CI	*P*-value	OR	95% CI	*P*-value
FMR cohort (*n* = 1333)						
BMI 18.5 or higher	0.907	0.665–1.238	.540			
BNP median or higher	1.113	0.832–1.487	.471			
NT-pro-BNP median or higher	1.000	1.000–1.000	.189			
LVEF (per 1.0% increase)	0.997	0.988–1.005	.443			
LVEDV	1.003	1.001–1.004	.003	1.003	1.001–1.005	.012
LAVI	1.005	1.003–1.008	<.001	1.004	1.001–1.007	.005
MV mean PG	1.000	1.000–1.000	.383			
TRPG	1.003	0.994–1.013	.460			
Stroke volume	0.991	0.983–0.999	.023	0.989	0.980–0.998	.015
MR EROA	4.725	2.052–10.881	<.001	2.111	0.752–5.926	.156
MV orifice area	1.080	0.989–1.176	.087			
TAPSE	0.986	0.972–1.001	.569			
E wave	1.002	0.999–1.005	.321			
Septal E/e′	0.986	0.972–1.001	.073			
Grasping length of AML	0.983	0.954–1.012	.243			
Grasping length of PML	0.998	0.957–1.040	.073			
Tenting height	0.971	0.934–1.009	.139			
Coaptation length	0.962	0.881–1.050	.386			
Coaptation gap	1.007	0.670–1.514	.973			
Calcification of target area	0.760	0.374–1.546	.449			
G2 device use	0.987	0.772–1.261	.914			
Long clip use	1.148	0.792–1.662	.466			
Wide clip use	0.907	0.678–1.212	.510			
Number of clips	1.351	1.040–1.754	.024	1.084	0.784–1.499	.625
DMR cohort (*n* = 532)						
BMI 18.5 or higher	0.769	0.503–1.174	.224			
BNP median or higher	0.679	0.443–1.042	.077			
NT-pro-BNP median or higher	0.853	0.520–1.399	.529			
LVEF	1.014	0.997–1.031	.108			
LVEDV	1.001	0.997–1.005	.624			
LVESV	1.000	0.994–1.007	.995			
LAVI	1.006	1.001–1.011	.017	1.003	0.996–1.009	.393
MV mean PG	1.094	0.939–1.274	.250			
TRPG	1.014	1.002–1.027	.025	1.006	0.989–1.023	.516
Stroke volume	0.988	0.976–1.000	.047	0.988	0.972–1.003	.124
EROA	3.379	1.394–8.188	.007	0.230	0.033–1.616	.140
MR regurgitation volume	1.009	1.003–1.016	.006	1.017	1.003–1.032	.018
MV orifice area	0.981	0.863–1.116	.774			
TAPSE	1.002	0.963–1.043	.905			
E wave	1.000	0.997–1.003	.769			
Septal E/e′	0.997	0.980–1.015	.760			
Grasping length of AML	1.008	0.996–1.053	.707			
Grasping length of PML	1.038	0.985–1.095	.162			
Prolapse gap	1.158	1.067–1.257	<.001	1.157	1.049–1.277	.003
Prolapse width	1.034	0.983–1.088	.190			
Flail valve	1.071	0.693–1.655	.758			
Calcification of target area	0.619	0.283–1.352	.229			
Generation of device	1.11	0.78–1.59	.570			
Central jet or not	0.792	0.55–1.15	.222			
Jet direction	1.000	1.000–1.000	.551			
Bileaflet prolapse	1.225	0.780–1.923	.379			
Chordal rupture	1.266	0.884–1.812	.198			
G2 device use	1.109	0.776–1.586	.570			
Long clip use	0.759	0.448–1.286	.306			
Wide clip use	0.735	0.469–1.153	.180			
Number of clips	0.923	0.649–1.313	.657			

Abbreviations as seen in *Tables 1* and *2*. AML, anterior mitral leaflet; PML, posterior mitral leaflet.

### Serial changes of clinical parameters from baseline to 1 year after M-TEER

**Figure 4:**
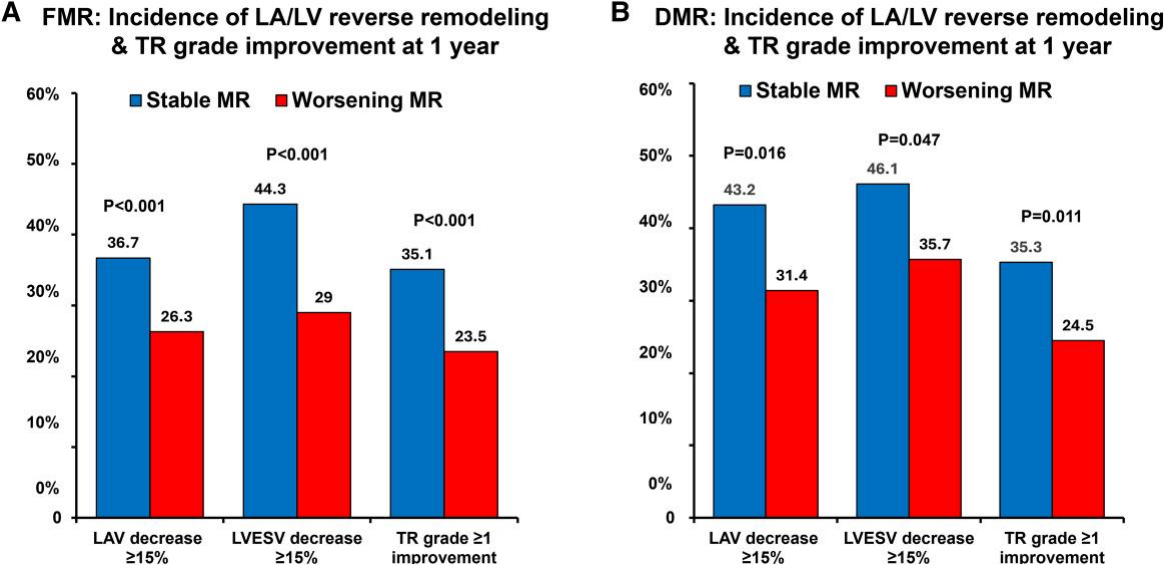
The incidence of left atrial/left ventricular reverse remodelling and tricuspid regurgitation grade improvement from baseline to 1 year after mitral transcatheter edge-to-edge repair. In patients with functional mitral regurgitation, all parameters showed significantly greater improvement in the stable mitral regurgitation group compared to the worsening mitral regurgitation group (*A*). In patients with degenerative mitral regurgitation, the same results were confirmed (*B*). LAV, left atrial volume; LVEF, left ventricle ejection fraction; LVESV, left ventricle end-systolic volume

Between the stable MR and worsening MR groups, the serial changes of several parameters are presented in *Table 6*. In the overall cohort, the Δ values of BNP, NT-pro-BNP, LAV, LAVI, LVEF, LVESV, and ΔTRPG were significantly different in the two groups (all *P* < .05). The improvement in NYHA class ≤2 at 1 year was more prevalent in the stable MR group than in the worsening MR group (96.3% vs 92.7%, *P* = .003). When stratified by aetiology, all comparisons were significant in FMR (all *P* < .05). In DMR, differences were directionally similar but not significant for NYHA class ≤2, BNP, LVEF, and TRPG. In terms of LA and LV reverse remodelling and TR grade improvement, were more prevalent in the stable MR group than those of the worsening group (Central Illustration). These results were consistently observed regardless of the difference between FMR and DMR (*Figure 4*).

**Table 6:** Changes of clinical parameters from baseline to 1 year after mitral transcatheter edge-to-edge repair

	*N*	Stable MR group	Worsening MR group	*P*-value
Overall cohort				
NYHA2 or less	1615	1185 (96.3%)	430 (92.7%)	.003
Δ BNP, pg/ml	1198	−37.9 (−356.5 to 88.0)	38.8 (−95.8 to 163.4)	<.001
Δ NT-pro-BNP, pg/ml	730	0.0 (−935.0 to 837.0)	507.2 (−619.0 –3247.2)	<.001
Δ LAV, ml	1621	−9.0 (−27.0 to 11.0)	−3.0 (−21.5 to 18.0)	<.001
Δ LAVI, ml/m^2^	1601	−6.3 (−18.7 to 6.7)	−1.8 (−15.1 to 11.1)	<.001
Δ LVEF, %	1538	−0.1 (−5.2 to 6.4)	−1.8 (−7.0 to 3.5)	<.001
Δ LVESV, ml	1545	−6.0 (−23.0 to 5.0)	−2.0 (−14.9 to 10.0)	<.001
Δ TRPG, mmHg	1692	−3.0 (−13.0 to 3.5)	−3.0 (−14.0 to 6.0)	<.001
FMR cohort				
NYHA2 or less	907	867 (95.6%)	273 (91.0%)	.005
Δ BNP, pg/ml	872	−36.7 (−386.4 to 90.6)	42.6 (−140.9 to 207.4)	<.001
Δ NT-pro-BNP, pg/ml	520	23.0 (−943.8 –1149.5)	447.6 (−683.8 to 3280.8)	.003
Δ LAV, ml	1145	−8.0 (−26.0 to 11.5)	−1.0 (−20.8 to 21.0)	<.001
Δ LAVI, ml/m^2^	1141	−5.6 (−17.6 to 7.1)	−0.6 (−14.2 to 13.3)	<.001
Δ LVEF, %	1128	0.8 (−4.5 to 7.4)	−1.0 (−6.7 to 4.0)	<.001
Δ LVESV, ml	1135	−7.0 (−28.0 to 6.3)	−1.0 (−18.0 to 14.0)	<.001
Δ TRPG, mmHg	1208	−3.0 (−13.0 to 3.0)	−0.5 (−4.8 to 9.8)	<.001
DMR cohort				
NYHA2 or less	487	318 (98.5%)	157 (95.7%)	.117
Δ BNP, pg/ml	212	−27.5 ( −207.3 to 29.7)	1.0 ( −123.4 to 97.2)	.252
Δ NT-pro-BNP, pg/ml	140	−25.5 (−535.8 to 385.0)	186.0 ( −434.5 to 977.2)	.003
Δ LAV, ml	466	−11.0 (−31.5 to 8.0)	−5.0 (−22.5 to 11.0)	.025
Δ LAVI, ml/m^2^	460	−8.1 (−22.5 to 5.5)	−4.3 (−16.9 to 7.5)	.028
Δ LVEF, %	410	−2.6 (−7.4 to 2.3)	−3.2 (−8.5 to 2.3)	.435
Δ LVESV, ml	410	−5.0 (−13.0 to 1.0)	−2.0 (−11.0 to 4.0)	.011
Δ TRPG, mmHg	484	−4.0 (−12.0 to 3.0)	−1.2 (−11.0 –7.0)	.103

Abbreviations as seen in *Tables 1* and *2*.

## Discussion

The main findings of the current study are summarized in the Central Illustration. Among patients who achieved mild MR after M-TEER and survived for 1 year, 28.3% experienced MR worsening. The prevalence of MR worsening was ∼10% higher in DMR patients than in FMR patients. The predictive factors for MR worsening differed between FMR and DMR, emphasizing the importance of assessing these clinical factors before M-TEER. In contrast, those who maintained stable MR exhibited multiple clinical benefits, including symptom relief, serum BNP and/or NT-pro-BNP decrease, and several better echocardiographic findings at 1 year.

### Predictive factors of MR worsening at 1 year after M-TEER

The presence of MR worsening at 1 year was identified as an independent risk factor for poor prognosis, demonstrating the critical role of keeping MR mild at the chronic phase of M-TEER. Even among patients who achieved almost perfect procedural results with mild or less MR immediately after M-TEER, there remains a risk of MR progression within 1 year. DMR patients were more prone to MR deterioration than FMR patients. This distinction highlights the need for aetiology-specific risk stratification before M-TEER. In FMR, larger LAVI, larger LV volumes, and lower stroke volume were associated with MR worsening, whereas in DMR, greater regurgitant volume and a wider prolapse gap—i.e. anatomic severity—were key. These patterns are partly consistent with prior reports on MR recurrence after M-TEER.^2,11,12^ The chamber remodelling is the dominant driver of FMR recurrence after M-TEER. In ventricular FMR, LV enlargement and LA dilation plausibly sustain tethering and transmit higher filling pressures, predisposing to recurrence. In atrial FMR, the signal was limited to stroke volume and should be interpreted cautiously given the sample size. Overall, the data supports that FMR patients with advanced adverse remodelling (larger LA/LV) are relatively difficult to maintain a lower MR grade after M-TEER. The use of the latest G4 MitraClip device did not reduce the incidence of worsening MR in this study. Although G4 MitraClip has been linked to improved procedural metrics in prior studies,^13,14^ our adjusted analyses focusing on 1-year MR recurrence did not identify device generation, clip number, or clip size as independent predictors. Differences in endpoints, potential confounding by indication, and limited power for subgroup contrasts, together with the predominant role of etiologic/anatomic substrate, may explain these findings.

### Clinical significance of MR stability after M-TEER

A key issue in this study is that the residual MR grade was assessed semi-quantitatively at each institution. When diving into mild MR and other groups, favourable outcomes were consistently observed across multiple parameters, including patient symptoms, blood examinations, and numerous echocardiographic findings at the 1-year follow-up. The improvement in NYHA classification was more pronounced. The BNP and NT-pro-BNP values, which are established biomarkers for HF severity and prognosis, showed a significant decrease. The cut-off for LA reverse remodelling was set at a 15% volume reduction in LAV.^8^ In addition to the significant decrease in absolute values of LAV and LAVI, substantial LA reverse remodelling was observed. There are multiple echocardiographic definitions for LV reverse remodelling, while the current study applied the most commonly used value of reduction with at least 15% in LVESV.^9,10^ In addition to a reduction in LVESV, improvement in LVEF was also observed, suggesting that LV reverse remodelling would likely be evident even if alternative parameters were used. In DMR patients, LVEF was largely preserved resulting in no difference in ΔEF between the two groups; however, LV reverse remodelling of LVESV was consistently observed. Previous pivotal data have reported that a reduction in LVEDV and LA volumes was associated with the degree of residual MR at 1 year in both FMR and DMR, while LVESV was significantly reduced in FMR but not in DMR.^15^ In our study, a significant reduction in all these parameters was observed. The larger sample size may have contributed to the stability of our results. In addition, the ΔTRPG was significantly lower and the TR grade improvement was higher. Stable MR is expected to prevent or reverse remodelling of LA and LV, with possible positive effects on the right ventricular function. In contrast, the worsening MR group has also confirmed the LA and LV reverse remodelling, largely driven by a shift towards moderate MR, which suggests that the reduction in MR severity from baseline is responsible for these improvements.

### Patient selection and management to maintain stable MR after M-TEER

Procedural success of M-TEER is generally defined as maintaining an MR grade of moderate or less, while true treatment success may be better defined as achieving and keeping a lower MR grade in the chronic phase. In our cohort, worsening MR at 1 year after M-TEER was linked to adverse prognosis, including all-cause death or HF rehospitalization, in a landmark analysis. Notably, most cases of worsening MR were moderate (2+), whereas a smaller subset (3.6% overall; 12.9% of the worsening group) progressed to MR ≥3+. Although approximately one-third of patients with MR ≥3+ underwent repeat intervention (repeat M-TEER or surgery) when symptomatic, their outcomes remained unfavourable. These results highlight the clinical importance of preventing significant MR recurrence and early identification of patients at risk for deterioration after M-TEER. The variables linked to MR recurrence in our study align with the patient selection principles from the pivotal Cardiovascular Outcomes Assessment of the MitraClip Percutaneous Therapy for Heart Failure Patients with Functional Mitral Regurgitation (COAPT) randomized trial,^2^ which demonstrated benefit under stringent criteria that excluded markedly dilated ventricles. In exploratory (non-displayed) analyses, only ∼10% of real-world patients met a COAPT-like profile, yet those patients showed more durable MR reduction. These observations support that meticulous patient selection—avoiding advanced adverse remodelling and ensuring anatomic suitability—is a key lever for sustaining lower MR after M-TEER. Beyond M-TEER itself, sustaining stable MR depends on optimizing guideline-directed HF therapy and strict blood pressure/volume control. In addition, targeted management of atrial fibrillation (including ablation when appropriate) and implantation or re-optimization of cardiac electronic devices may be considered in eligible patients to mitigate recurrence. The results of our study will provide valuable insights into decision-making indications of M-TEER or adjunctive transcatheter annuloplasty in addition to M-TEER, alternative catheter-based therapies, such as transcatheter mitral valve replacement, and cardiac surgery within the heart team.

### Limitations

This study has several limitations. First, this is a retrospective study, in which unpredictive factors or bias for patient selection may have affected the results. Second, patients who died and lost follow-up within 1 year after M-TEER are excluded. These may bias the findings, especially in the analysis for predictors of worsening MR after the procedure. However, this was a deliberate design choice, as the primary aim of the study was to evaluate the long-term maintenance of MR reduction and to identify predictors of its durability. Third, post-procedure medical therapy optimization could influence the dynamic trajectory of MR, while this was not examined in this study because it requires time-varying, longitudinal modelling; this remains a focus for future work. Fourth, residual MR is expected to fluctuate over time, with potential improvement or worsening during the follow-up period. As MR severity was assessed only at discharge and at the 1-year follow-up, dynamic changes in MR may not have been fully captured. However, this limitation reflects the inherent constraints of available time points in clinical follow-up. Finally, as described in the Discussion section, post-operative MR assessment is often based on qualitative assessment alone in actual clinical practice, and this assessment seems to be in line with reality. These are also evaluated by individual centre echo-lab without central core-lab analysis. Before data were enrolled in the OCEAN-Mitral registry, the investigators decided to use a consensus document based on the guidelines and share it with the participating institutions to minimize the knowledge and technical gap. However, further research is required to validate these findings.

## Conclusions

Approximately one-fourth to one-third of patients who initially achieved MR ≤ mild after M-TEER experienced a worsening of MR within the first year. The MR worsening group was found to have a lower survival rate beyond 1 year after M-TEER, underscoring that maintaining MR ≤ mild in the chronic phase is critical. In addition, the MR worsening group had more significant symptoms and higher BNP levels during follow-up. Key anatomical and functional differences—such as large LAVI, lower stroke volume, and short tenting height in FMR, and large regurgitant volume or a wide prolapse gap in DMR—may underlie differential responses to therapy and represent potential risk factors for worsening MR in the chronic phase. Accurate risk assessment and efforts to maintain MR as less as possible in the chronic phase are required to maximize treatment efficacy and improve the long-term prognosis of patients undergoing M-TEER.

## Supplementary Material

xvag016_Supplementary_Data

## Declarations

### Data Availability

The data underlying this article will be shared on reasonable request to the corresponding author.

### Pre-registered Clinical Trial Number

None supplied.
