# Supplementary material for: Impact of maintaining mild mitral regurgitation beyond 1 year after mitral transcatheter edge-to-edge repair
Source: ESC Heart Fail. 2026 Jan 14;13(2):xvag016. doi: 10.1093/eschf/xvag016 (PMC13108316; doi:10.1093/eschf/xvag016)
Supplement: xvag016_Supplementary_Data [file xvag016_supplementary_data.docx]

**Table 1. Patient characteristics.**

|  | N | Stable MR group (n=1336) | Worsening MR group (n=529) | *p value* |
| --- | --- | --- | --- | --- |
| Baseline clinical characteristics |  |  |  |  |
| Age, years | 1,865 | 78.2 ± 9.5 | 78.6 ± 9.6 | 0.354 |
| Male, n (%) | 1,865 | 741 (55.5%) | 279 (52.7%) | 0.287 |
| Height, cm | 1,865 | 157.3 ± 10.1 | 156.7 ± 10.0 | 0.205 |
| Weight, kg | 1,865 | 53.8 ± 11.8 | 52.4 ± 11.2 | 0.020 |
| BMI, kg/m^2^ | 1,865 | 21.6 ± 3.5 | 21.2 ± 3.4 | 0.032 |
| Comorbidities and general status |  |  |  |  |
| Systolic BP, mmHg | 1,865 | 111.8 ± 19.0 | 110.5 ± 17.9 | 0.155 |
| Diastolic BP, mmHg | 1,865 | 64.9 ± 12.9 | 64.6 ± 12.2 | 0.683 |
| Heart rate, /min. | 1,865 | 73 ± 15 | 74 ± 14 | 0.302 |
| Hypertension, n (%) | 1,865 | 899 (67.3%) | 348 (65.8%) | 0.533 |
| Dyslipidemia, n (%) | 1,865 | 680 (50.9%) | 262 (49.5%) | 0.594 |
| Diabetes mellitus, n (%) | 1,865 | 370 (27.7%) | 113 (21.4%) | 0.005 |
| Smoking, n (%) | 1,865 | 453 (33.9%) | 174 (32.9%) | 0.640 |
| Chronic kidney disease, n (%) | 1,865 | 1136 (85.0%) | 457 (86.4%) | 0.453 |
| Dialysis, n (%) | 1,865 | 73 (5.5%) | 23 (4.3%) | 0.325 |
| COPD, n (%) | 1,865 | 100 (7.5%) | 54 (10.2%) | 0.054 |
| Coronary artery disease, n (%) | 1,865 | 487 (36.5%) | 167 (31.6%) | 0.046 |
| Atrial fibrillation, n (%) | 1,865 | 788 (59.0%) | 327 (61.8%) | 0.272 |
| NYHA class III or IV, n (%) | 1,865 | 816 (61.0%) | 292 (55.2%) | 0.020 |
| Clinical frailty scale ≥4, n (%) | 1,787 | 641 (49.6%) | 239 (48.3%) | 0.615 |
| Prior HF admission, n (%) | 1,812 | 1.44 ± 1.1 | 1.44 ± 1.1 | 0.990 |
| Prior HF within 1 year, n (%) | 1,861 | 1.03 ± 1.0 | 1.00 ± 0.9 | 0.488 |
| EuroSCORE II, % | 1,599 | 6.5 ± 5.8 | 6.3 ± 6.0 | 0.535 |
| STS score for MV repair, % | 1,414 | 6.0 [3.4 – 9.6] | 5.6 [3.3 – 9.2] | 0.322 |
| STS score for MV replacement, % | 1,751 | 8.5 [5.3 – 12.7] | 8.6 [5.6 – 13.3] | 0.637 |
| Laboratory data |  |  |  |  |
| BNP, pg/ml | 1,354 | 319.2 [160.7 – 638.4] | 290.3 [155.8 – 593.3] | 0.292 |
| NT-proBNP, pg/ml | 1,003 | 2,292 [1,050 – 4,585] | 2,192 [821 – 4,386] | 0.235 |
| Hemoglobin, g/dl | 1,865 | 11.9 ± 1.9 | 11.8 ± 1.7 | 0.411 |
| Albumin, g/dl | 1,857 | 3.7 ± 0.5 | 3.47 ± 0.5 | 0.441 |
| Creatinine, mg/dl | 1,856 | 1.6 ± 1.4 | 1.6 ± 1.5 | 0.682 |
| eGFR, [mL/min/1.73m^2^](https://www.bing.com/ck/a?!&&p=fcf60d6224d4a3f8JmltdHM9MTcwNDA2NzIwMCZpZ3VpZD0yYWY5MzQ1Yi04OTdhLTY0OGEtMzczOC0yNTU0ODg5NDY1MWImaW5zaWQ9NTY3OA&ptn=3&ver=2&hsh=3&fclid=2af9345b-897a-648a-3738-25548894651b&psq=egfr+%e5%8d%98%e4%bd%8d&u=a1aHR0cHM6Ly9waGFybWFjaXN0YS5qcC9jb250ZW50cy9za2lsbHVwL2luc3BlY3Rpb24va2lkbmV5LzQ0NTkv&ntb=1) | 1,856 | 40.3 ± 19.8 | 40.8 ± 18.4 | 0.598 |
| Sodium, mEq/l | 1,863 | 139.3 ± 3.3 | 139.3 ± 3.5 | 0.989 |
| Medication |  |  |  |  |
| Loop diuretics, n (%) | 1,865 | 1062 (79.5%) | 426 (80.5%) | 0.654 |
| Tolvaptan, n (%) | 1,865 | 532 (39.8%) | 207 (39.1%) | 0.791 |
| ARNI, n (%) | 1,864 | 113 (8.5%) | 43 (8.1%) | 0.853 |
| Beta-blocker, n (%) | 1,833 | 1021 (77.8%) | 373 (71.7%) | 0.008 |
| MRA, n (%) | 1,862 | 738 (55.3%) | 282 (53.5%) | 0.502 |
| SGLT2 inhibitor, n (%) | 1,865 | 229 (17.1%) | 81 (15.3%) | 0.370 |

Values are numbers (%) or mean ± SD. ARNI, angiotensin receptor neprilysin inhibitor; BMI, body mass index; BNP, B-type natriuretic peptide; BP, blood pressure; COPD, chronic obstructive pulmonary disease; eGFR, estimated glomerular filtration ratio; HF, heart failure; MRA, mineralocorticoid receptor antagonist; MV, mitral valve; NYHA, New York Heart Association; SGLT-2, sodium glucose co-transporter-2; STS score, Society of Thoracic Surgeons score.

## **Table 2. Echocardiographic parameters and procedural variables of study patients.**

|  | N | Stable MR group (n=1336) | Worsening MR group (n=529) | *p value* |
| --- | --- | --- | --- | --- |
| Echo parameters before procedure |  |  |  |  |
| LVEF, % | 1,865 | 44.3 ± 16.1 | 46.2 ± 17.1 | 0.023 |
| LVDd, mm | 1,865 | 56.4 ± 9.9 | 57.3 ± 10.6 | 0.089 |
| LVDs, mm | 1,865 | 43.8 ± 13.0 | 44.0 ± 14.0 | 0.843 |
| LVEDV, ml | 1,790 | 143.8 ± 66.7 | 149.8 ± 69.6 | 0.091 |
| LVESV, ml | 1,733 | 89.0 ± 58.9 | 92.2 ± 64.2 | 0.325 |
| LAVI, ml/m^2^ | 1,811 | 46.3 ± 1.3 | 47.8 ± 2.1 | <0.001 |
| TRPG, mmHg | 1,815 | 33.3 ± 13.5 | 34.9 ± 14.8 | 0.027 |
| TAPSE, mm | 1,528 | 16.5 ± 4.8 | 16.7 ± 4.6 | 0.532 |
| Mitral E wave, cm/s | 1,801 | 98.7 ± 51.0 | 102.9 ± 34.6 | 0.090 |
| e’ (septal), cm/s | 1,699 | 5.30 ± 2.4 | 5.67 ± 2.2 | 0.004 |
| E/e' (septal), cm/s | 1,294 | 17.5 ± 8.6 | 16.6 ± 6.5 | 0.060 |
| MR EROA, cm^2^ | 1,675 | 0.35 ± 0.2 | 0.41 ± 0.2 | <0.001 |
| MR regurgitant volume, ml | 1,734 | 52.7 ± 24.3 | 59.0 ± 28.0 | <0.001 |
| MR regurgitant fraction, % | 1,287 | 48.4 ± 18.4 | 52.0 ± 16.9 | 0.002 |
| MV orifice area, cm^2^ | 1,492 | 5.2 ± 1.5 | 5.3 ± 1.7 | 0.195 |
| MV mean PG, mmHg | 1,521 | 1.8 ± 1.1 | 1.9 ± 1.1 | 0.100 |
| AS ≥moderate, n (%) | 1,780 | 42 (3.3%) | 12 (2.4%) | 0.359 |
| AR ≥moderate, n (%) | 1,861 | 123 (9.2%) | 52 (9.8%) | 0.725 |
| TR ≥moderate, n (%) | 1,861 | 421 (31.6%) | 195 (36.9%) | 0.033 |
| Pathogenesis of MR |  |  |  |  |
| FMR | 1,333 | 988 (74.0%) | 345 (65.2%) | <0.001 |
| DMR | 532 | 348 (26.0%) | 184 (34.8%) |  |
| Pathogenesis of DMR | 532 |  |  |  |
| Bileaflet prolapse, n (%) | 532 | 61 (17.5%) | 38 (20.7%) | 0.413 |
| Chordal rupture, n (%) | 532 | 148 (42.5%) | 89 (48.4%) | 0.201 |
| Central regurgitation, n (%) | 532 | 136 (39.1%) | 62 (33.7%) | 0.258 |
| Valve flail, n (%) | 427 | 80 (28.8%) | 45 (30.2%) | 0.824 |
| Jet direction | 392 |  |  |  |
| Central | 114 | 74 (28.2%) | 40 (30.8%) | 0.874 |
| Anterior | 150 | 104 (39.7%) | 46 (35.4%) |  |
| Posterior | 99 | 65 (24.8%) | 34 (26.2%) |  |
| Others | 29 | 19 (7.3%) | 10 (7.7%) |  |
| Pathogenesis of FMR | 1333 |  |  |  |
| Atrial functional | 300 | 224 (16.8%) | 76 (14.4%) | 0.209 |
| Ventricular Functional | 1,033 | 764 (77.3%) | 269 (78.0%) | 0.823 |
| Coaptation gap, mm | 79 | 2.6 ± 1.1 | 1.6 ± 1.4 | 0.974 |
| Coaptation length, mm | 891 | 3.0 ± 2.3 | 2.9 ± 1.3 | 0.383 |
| Tenting height, mm | 1036 | 7.8 ± 3.9 | 7.4 ± 3.6 | 0.139 |
| Procedural variables |  |  |  |  |
| G2 device use, n (%) | 1865 | 638 (47.8%) | 256 (48.4%) | 0.837 |
| Long clip if used 1 clip, n (%) | 1,332 | 189 (19.0) | 73 (20.2) | 0.781 |
| Wide clip if used 1 clip, n (%) | 1,332 | 475 (49.0) | 162 (44.8) | 0.170 |
| Number of clip, n | 1,865 | 1.28 ± 0.5 | 1.33 ± 0.5 | 0.056 |
| Post MV mean PG, mmHg | 1744 | 2.9 ± 1.9 | 2.9 ± 1.4 | 0.795 |

Values are numbers (%) or mean ± SD. AR, aortic regurgitation; AS, aortic stenosis; DMR, degenerative mitral regurgitation; EROA, effective regurgitant orifice area; FMR, functional mitral regurgitation; G2, generation 2; MR, mitral regurgitation; MV, mitral valve; PG, pressure gradient; TAPSE, tricuspid annular plane systolic excursion; TR, tricuspid regurgitation; TRPG, tricuspid regurgitation pressure gradient; LAVI, left atrial volume index; LVDd, left ventricular end-diastolic diameter; LVDs, left ventricular end-systolic diameter; LVEDV, left ventricular end-diastolic volume; LVEF, left ventricular ejection fraction; LVESV, left ventricular end-systolic volume.

**Table 3. Cox regression multivariable models of the association between composite clinical outcomes and clinical variables.**

|  | Univariable analysis | | | Multivariable analysis | | |
| --- | --- | --- | --- | --- | --- | --- |
| Overall cohort (n=1865) | HR | 95% CI | p value | HR | 95% CI | p value |
| MR severity 1 year after M-TEER |  | | | | | |
| Worsening MR (for Stable MR) | 1.56 | 1.20-2.04 | 0.001 | 1.80 | 1.30-2.49 | <0.001 |
| Clinical variables |  |  |  |  |  |  |
| Age, years | 1.01 | 0.99-1.02 | 0.374 | 1.01 | 0.99-1.03 | 0.605 |
| Male | 1.13 | 0.87-1.47 | 0.351 | 1.02 | 0.72-1.42 | 0.932 |
| BMI, kg/m^2^ | 0.95 | 0.91-0.99 | 0.009 | 0.99 | 0.94-1.04 | 0.580 |
| Systolic BP, mmHg | 0.99 | 0.99-1.00 | 0.028 | 0.99 | 0.99-1.01 | 0.361 |
| NYHA class III/IV | 1.60 | 1.21-2.11 | 0.001 | 1.22 | 0.85-1.74 | 0.281 |
| Clinical frailty scale ≥4 | 1.35 | 1.04-1.76 | 0.026 | 1.15 | 0.81-1.63 | 0.430 |
| Euro Score II | 1.04 | 1.02-1.06 | <0.001 | 1.01 | 0.98-1.03 | 0.672 |
| Albumin, g/dl | 0.58 | 0.45-0.73 | <0.001 | 0.80 | 0.56-1.12 | 0.192 |
| Hemoglobin, mg/dl | 0.87 | 0.81-0.94 | <0.001 | 0.96 | 0.86-1.06 | 0.415 |
| Sodium, mEq/l | 0.96 | 0.93-0.99 | 0.021 | 0.98 | 0.94-1.02 | 0.363 |
| eGFR, [mL/min/1.73m^2^](https://www.bing.com/ck/a?!&&p=fcf60d6224d4a3f8JmltdHM9MTcwNDA2NzIwMCZpZ3VpZD0yYWY5MzQ1Yi04OTdhLTY0OGEtMzczOC0yNTU0ODg5NDY1MWImaW5zaWQ9NTY3OA&ptn=3&ver=2&hsh=3&fclid=2af9345b-897a-648a-3738-25548894651b&psq=egfr+%e5%8d%98%e4%bd%8d&u=a1aHR0cHM6Ly9waGFybWFjaXN0YS5qcC9jb250ZW50cy9za2lsbHVwL2luc3BlY3Rpb24va2lkbmV5LzQ0NTkv&ntb=1) | 0.99 | 0.98-0.99 | <0.001 | 0.99 | 0.99-1.00 | 0.206 |
| Hypertension | 1.01 | 0.77-1.34 | 0.921 |  |  |  |
| Diabetes mellitus | 1.12 | 1.01-1.32 | 0.029 | 1.14 | 0.81-1.62 | 0.447 |
| COPD | 1.35 | 0.91-2.02 | 0.139 |  |  |  |
| Dialysis dependent | 1.27 | 0.72-2.22 | 0.407 |  |  |  |
| Atrial fibrillation/flutter | 1.08 | 0.93-1.24 | 0.336 |  |  |  |
| Previous stroke | 1.03 | 0.68-1.55 | 0.897 |  |  |  |
| LVEF, % | 0.99 | 0.98-0.99 | <0.001 | 0.99 | 0.98-1.01 | 0.306 |
| LAVI, ml/m^2^ | 1.00 | 1.00-1.00 | 0.177 |  |  |  |
| MV mean PG, mmHg | 1.00 | 1.00-1.00 | 0.536 |  |  |  |
| TRPG, mmHg | 1.00 | 0.99-1.01 | 0.401 |  |  |  |
| TAPSE, mm | 0.96 | 0.93-0.99 | 0.012 | 0.98 | 0.95-1.02 | 0.258 |
| Stroke volume, ml | 1.00 | 0.99-1.01 | 0.607 |  |  |  |
| MR EROA, cm^2^ | 1.06 | 0.49-2.28 | 0.886 |  |  |  |
| MR regurgitation volume, ml | 1.00 | 0.99-1.00 | 0.378 |  |  |  |
| MV orifice area, cm^2^ | 1.00 | 1.00-1.00 | 0.176 |  |  |  |
| FMR | 2.32 | 1.62-3.33 | <0.001 | 1.87 | 1.13-3.10 | 0.014 |
| G2 clip use | 0.84 | 0.65-1.09 | 0.197 |  |  |  |
| Long Clip when used 1 clip | 0.79 | 0.46-1.35 | 0.393 |  |  |  |
| Wide clip when used 1 clip | 1.03 | 0.75-1.42 | 0.848 |  |  |  |

Abbreviations as seen in Table 1 and Table 2

**Table 4. Cox regression multivariable models of the association between all-cause mortality and clinical variables**

|  | Univariable analysis | | | Multivariable analysis | | |
| --- | --- | --- | --- | --- | --- | --- |
| Overall cohort (n=1865) | HR | 95% CI | p value | HR | 95% CI | p value |
| MR severity 1 year after M-TEER |  |  |  |  |  |  |
| Worsening MR (for Stable MR) | 1.67 | 1.15-2.40 | 0.007 | 1.79 | 1.12-2.87 | 0.015 |
| Clinical variables |  |  |  |  |  |  |
| Age, years | 1.02 | 1.00-1.04 | 0.108 | 1.02 | 0.99-1.05 | 0.322 |
| Male | 1.44 | 0.98-2.10 | 0.060 | 1.69 | 1.02-2.80 | 0.044 |
| BMI, kg/m^2^ | 0.90 | 0.85-0.95 | <0.001 | 0.91 | 0.84-0.99 | 0.020 |
| Systolic BP, mmHg | 0.99 | 0.98-0.99 | 0.027 | 0.99 | 0.98-1.01 | 0.349 |
| NYHA class III/IV | 1.01 | 0.69-1.50 | 0.945 |  |  |  |
| Clinical frailty scale ≥4 | 1.01 | 0.83-1.23 | 0.921 |  |  |  |
| Euro Score II | 1.78 | 0.90-3.51 | 0.096 |  |  |  |
| Albumin, g/dl | 1.24 | 1.01-1.52 | 0.045 | 1.16 | 0.69-1.94 | 0.581 |
| Hemoglobin, mg/dl | 2.00 | 1.33-3.01 | 0.001 | 1.19 | 0.70-2.02 | 0.522 |
| Sodium, mEq/l | 1.64 | 1.14-2.38 | 0.009 | 1.43 | 0.86-2.36 | 0.168 |
| eGFR, [mL/min/1.73m^2^](https://www.bing.com/ck/a?!&&p=fcf60d6224d4a3f8JmltdHM9MTcwNDA2NzIwMCZpZ3VpZD0yYWY5MzQ1Yi04OTdhLTY0OGEtMzczOC0yNTU0ODg5NDY1MWImaW5zaWQ9NTY3OA&ptn=3&ver=2&hsh=3&fclid=2af9345b-897a-648a-3738-25548894651b&psq=egfr+%e5%8d%98%e4%bd%8d&u=a1aHR0cHM6Ly9waGFybWFjaXN0YS5qcC9jb250ZW50cy9za2lsbHVwL2luc3BlY3Rpb24va2lkbmV5LzQ0NTkv&ntb=1) | 1.05 | 1.03-1.08 | <0.001 | 1.01 | 0.98-1.05 | 0.516 |
| Hypertension | 1.00 | 1.00-1.00 | 0.052 |  |  |  |
| Diabetes mellitus | 1.00 | 1.00-1.00 | 0.086 |  |  |  |
| COPD | 0.84 | 0.75-0.93 | 0.001 | 0.96 | 0.83-1.11 | 0.568 |
| Dialysis dependent | 0.39 | 0.29-0.54 | <0.001 | 0.67 | 0.41-1.10 | 0.114 |
| Atrial fibrillation/flutter | 0.99 | 0.98-1.00 | 0.150 |  |  |  |
| Previous stroke | 0.96 | 0.91-1.01 | 0.117 |  |  |  |
| LVEF, % | 0.98 | 0.97-0.99 | 0.005 | 1.00 | 0.98-1.02 | 0.814 |
| LAVI, ml/m^2^ | 1.00 | 1.00-1.00 | 0.217 |  |  |  |
| MV mean PG, mmHg | 1.00 | 0.98-1.01 | 0.730 |  |  |  |
| TRPG, mmHg | 0.95 | 0.90-0.99 | 0.012 | 0.97 | 0.92-1.02 | 0.236 |
| TAPSE, mm | 1.00 | 1.00-1.00 | 0.563 |  |  |  |
| Stroke volume, ml | 1.79 | 0.66-4.85 | 0.254 |  |  |  |
| MR EROA, cm^2^ | 1.00 | 0.99-1.01 | 0.908 |  |  |  |
| MR regurgitation volume, ml | 1.00 | 1.00-1.00 | 0.238 |  |  |  |
| MV orifice area, cm^2^ | 2.77 | 1.61-4.76 | <0.001 | 2.85 | 1.32-6.14 | 0.008 |
| FMR | 0.71 | 0.49-1.01 | 0.059 |  |  |  |
| G2 clip use | 0.77 | 0.35-1.68 | 0.511 |  |  |  |
| Long Clip when used 1 clip | 1.21 | 0.77-1.89 | 0.409 |  |  |  |

Abbreviations as seen in Table 1 and Table 2

**Table** **5. Predictive risk factors of worsening MR at 1-year following M-TEER**

| FMR cohort (n=1333) | Univariable analysis | | | Multivariable analysis | | |
| --- | --- | --- | --- | --- | --- | --- |
| Variables | OR | 95% CI | p value | OR | 95% CI | p value |
| BMI 18.5 or higher | 0.907 | 0.665-1.238 | 0.540 |  |  |  |
| BNP median or higher | 1.113 | 0.832-1.487 | 0.471 |  |  |  |
| NT-Pro BNP median or higher | 1.000 | 1.000-1.000 | 0.189 |  |  |  |
| LVEF (per 1.0% increase) | 0.997 | 0.988-1.005 | 0.443 |  |  |  |
| LVEDV | 1.003 | 1.001-1.004 | 0.003 | 1.003 | 1.001-1.005 | 0.012 |
| LAVI | 1.005 | 1.003-1.008 | <0.001 | 1.004 | 1.001-1.007 | 0.005 |
| MV mean PG | 1.000 | 1.000-1.000 | 0.383 |  |  |  |
| TRPG | 1.003 | 0.994-1.013 | 0.460 |  |  |  |
| Stroke volume | 0.991 | 0.983-0.999 | 0.023 | 0.989 | 0.980-0.998 | 0.015 |
| MR EROA | 4.725 | 2.052-10.881 | <0.001 | 2.111 | 0.752-5.926 | 0.156 |
| MV orifice area | 1.080 | 0.989-1.176 | 0.087 |  |  |  |
| TAPSE | 0.986 | 0.972-1.001 | 0.569 |  |  |  |
| E wave | 1.002 | 0.999-1.005 | 0.321 |  |  |  |
| Septal E/e’ | 0.986 | 0.972-1.001 | 0.073 |  |  |  |
| Grasping length of AML | 0.983 | 0.954-1.012 | 0.243 |  |  |  |
| Grasping length of PML | 0.998 | 0.957-1.040 | 0.073 |  |  |  |
| Tenting height | 0.971 | 0.934-1.009 | 0.139 |  |  |  |
| Coaptation length | 0.962 | 0.881-1.050 | 0.386 |  |  |  |
| Coaptation gap | 1.007 | 0.670-1.514 | 0.973 |  |  |  |
| Calcification of target area | 0.760 | 0.374-1.546 | 0.449 |  |  |  |
| G2 device use | 0.987 | 0.772-1.261 | 0.914 |  |  |  |
| Long clip use | 1.148 | 0.792-1.662 | 0.466 |  |  |  |
| Wide clip use | 0.907 | 0.678-1.212 | 0.510 |  |  |  |
| Number of clips | 1.351 | 1.040-1.754 | 0.024 | 1.084 | 0.784-1.499 | 0.625 |
| DMR cohort (n=532) |  |  |  |  |  |  |
| BMI 18.5 or higher | 0.769 | 0.503-1.174 | 0.224 |  |  |  |
| BNP median or higher | 0.679 | 0.443-1.042 | 0.077 |  |  |  |
| NT-Pro BNP median or higher | 0.853 | 0.520-1.399 | 0.529 |  |  |  |
| LVEF | 1.014 | 0.997-1.031 | 0.108 |  |  |  |
| LVEDV | 1.001 | 0.997-1.005 | 0.624 |  |  |  |
| LVESV | 1.000 | 0.994-1.007 | 0.995 |  |  |  |
| LAVI | 1.006 | 1.001-1.011 | 0.017 | 1.003 | 0.996-1.009 | 0.393 |
| MV mean PG | 1.094 | 0.939-1.274 | 0.250 |  |  |  |
| TRPG | 1.014 | 1.002-1.027 | 0.025 | 1.006 | 0.989-1.023 | 0.516 |
| Stroke volume | 0.988 | 0.976-1.000 | 0.047 | 0.988 | 0.972-1.003 | 0.124 |
| EROA | 3.379 | 1.394-8.188 | 0.007 | 0.230 | 0.033-1.616 | 0.140 |
| MR regurgitation volume | 1.009 | 1.003-1.016 | 0.006 | 1.017 | 1.003-1.032 | 0.018 |
| MV orifice area | 0.981 | 0.863-1.116 | 0.774 |  |  |  |
| TAPSE | 1.002 | 0.963-1.043 | 0.905 |  |  |  |
| E wave | 1.000 | 0.997-1.003 | 0.769 |  |  |  |
| Septal E/e’ | 0.997 | 0.980-1.015 | 0.760 |  |  |  |
| Grasping length of AML | 1.008 | 0.996-1.053 | 0.707 |  |  |  |
| Grasping length of PML | 1.038 | 0.985-1.095 | 0.162 |  |  |  |
| Prolapse gap | 1.158 | 1.067-1.257 | <0.001 | 1.157 | 1.049-1.277 | 0.003 |
| Prolapse width | 1.034 | 0.983-1.088 | 0.190 |  |  |  |
| Flail valve | 1.071 | 0.693-1.655 | 0.758 |  |  |  |
| Calcification of target area | 0.619 | 0.283-1.352 | 0.229 |  |  |  |
| Generation of device | 1.11 | 0.78-1.59 | 0.570 |  |  |  |
| Central jet or not | 0.792 | 0.55-1.15 | 0.222 |  |  |  |
| Jet direction | 1.000 | 1.000-1.000 | 0.551 |  |  |  |
| Bileaflet prolapse | 1.225 | 0.780-1.923 | 0.379 |  |  |  |
| Chordal rupture | 1.266 | 0.884-1.812 | 0.198 |  |  |  |
| G2 device use | 1.109 | 0.776-1.586 | 0.570 |  |  |  |
| Long clip use | 0.759 | 0.448-1.286 | 0.306 |  |  |  |
| Wide clip use | 0.735 | 0.469-1.153 | 0.180 |  |  |  |
| Number of clips | 0.923 | 0.649-1.313 | 0.657 |  |  |  |

Abbreviations as seen in Table 1 and Table 2

**Table 6. Changes of clinical parameters from baseline to 1 year after M-TEER.**

|  | N | Stable MR group | Worsening MR group | p value |
| --- | --- | --- | --- | --- |
| Overall cohort |  |  |  |  |
| NYHA2 or less | 1615 | 1185 (96.3%) | 430 (92.7%) | 0.003 |
| Δ BNP, pg/ml | 1198 | -37.9 [-356.5 – 88.0] | 38.8 [-95.8 – 163.4] | <0.001 |
| Δ NT-ProBNP, pg/ml | 730 | 0.0 [-935.0 – 837.0] | 507.2 [-619.0 –3247.2] | <0.001 |
| Δ LAV, ml | 1621 | -9.0 [-27.0 – 11.0] | -3.0 [-21.5 – 18.0] | <0.001 |
| Δ LAVI, ml/m^2^ | 1601 | -6.3 [-18.7 – 6.7] | -1.8 [-15.1 – 11.1] | <0.001 |
| Δ LVEF, % | 1538 | -0.1 [-5.2 – 6.4] | -1.8 [-7.0 – 3.5] | <0.001 |
| Δ LVESV, ml | 1545 | -6.0 [-23.0 – 5.0] | -2.0 [-14.9 – 10.0] | <0.001 |
| Δ TRPG, mmHg | 1692 | -3.0 [-13.0 – 3.5] | -3.0 [-14.0 – 6.0] | <0.001 |
| FMR cohort |  |  |  |  |
| NYHA2 or less | 907 | 867 (95.6%) | 273 (91.0%) | 0.005 |
| Δ BNP, pg/ml | 872 | -36.7 [-386.4 – 90.6] | 42.6 [-140.9 – 207.4] | <0.001 |
| Δ NT-ProBNP, pg/ml | 520 | 23.0 [-943.8 –1149.5] | 447.6 [-683.8 – 3280.8] | 0.003 |
| Δ LAV, ml | 1145 | -8.0 [-26.0 – 11.5] | -1.0 [-20.8 – 21.0] | <0.001 |
| Δ LAVI, ml/m^2^ | 1141 | -5.6 [-17.6 – 7.1] | -0.6 [-14.2 – 13.3] | <0.001 |
| Δ LVEF, % | 1128 | 0.8 [-4.5 – 7.4] | -1.0 [-6.7 – 4.0] | <0.001 |
| Δ LVESV, ml | 1135 | -7.0 [-28.0 – 6.3] | -1.0 [-18.0 – 14.0] | <0.001 |
| Δ TRPG, mmHg | 1208 | -3.0 [-13.0 – 3.0] | -0.5 [-4.8 – 9.8] | <0.001 |
| DMR cohort |  |  |  |  |
| NYHA2 or less | 487 | 318 (98.5%) | 157 (95.7%) | 0.117 |
| Δ BNP, pg/ml | 212 | -27.5 [ -207.3 – 29.7] | 1.0 [ -123.4 – 97.2] | 0.252 |
| Δ NT-ProBNP, pg/ml | 140 | -25.5 [-535.8 – 385.0] | 186.0 [ -434.5 – 977.2] | 0.003 |
| Δ LAV, ml | 466 | -11.0 [-31.5 – 8.0] | -5.0 [-22.5 – 11.0] | 0.025 |
| Δ LAVI, ml/m^2^ | 460 | -8.1 [-22.5 – 5.5] | -4.3 [-16.9 – 7.5] | 0.028 |
| Δ LVEF, % | 410 | -2.6 [-7.4 – 2.3] | -3.2 [-8.5 – 2.3] | 0.435 |
| Δ LVESV, ml | 410 | -5.0 [-13.0 – 1.0] | -2.0 [-11.0 – 4.0] | 0.011 |
| Δ TRPG, mmHg | 484 | -4.0 [-12.0 – 3.0] | -1.2 [-11.0 –7.0] | 0.103 |

Abbreviations as seen in Table 1 and Table 2

**Supplementary Table 1. Predictive risk factors of worsening MR at 1-year following M-TEER between the ventricular FMR and atrial FMR**

| Ventricular FMR cohort (n=1033) | Univariable analysis | | | Multivariable analysis | | |
| --- | --- | --- | --- | --- | --- | --- |
| Variables | OR | 95% CI | p value | OR | 95% CI | p value |
| BMI 18.5 or higher | 0.914 | 0.643-1.300 | 0.618 |  |  |  |
| BNP median or higher | 1.068 | 0.769-1.484 | 0.693 |  |  |  |
| NT-Pro BNP median or higher | 1.348 | 0.898-2.024 | 0.150 |  |  |  |
| LVEF (per 1.0% increase) | 0.991 | 0.977-1.006 | 0.250 |  |  |  |
| LVEDV | 1.003 | 1.001-1.005 | 0.002 | 1.003 | 1.000-1.005 | 0.020 |
| LAVI | 1.009 | 1.004-1.011 | <0.001 | 1.007 | 1.003-1.011 | <0.001 |
| MV mean PG | 1.000 | 1.000-1.000 | 0.216 |  |  |  |
| TRPG | 1.002 | 0.992-1.012 | 0.686 |  |  |  |
| Stroke volume | 0.994 | 0.985-1.003 | 0.187 |  |  |  |
| MR EROA | 4.528 | 1.793-11.44 | 0.001 | 2.418 | 0.827-7.071 | 0.107 |
| MV orifice area | 1.070 | 0.966-1.186 | 0.196 |  |  |  |
| TAPSE | 0.994 | 0.960-1.029 | 0.728 |  |  |  |
| E wave | 1.001 | 0.997-1.004 | 0.651 |  |  |  |
| Septal E/e’ | 0.986 | 0.970-1.002 | 0.092 |  |  |  |
| Grasping length of AML | 0.989 | 0.957-1.022 | 0.157 |  |  |  |
| Grasping length of PML | 1.011 | 0.965-1.060 | 0.644 |  |  |  |
| Tenting height | 0.970 | 0.929-1.013 | 0.164 |  |  |  |
| Coaptation length | 0.979 | 0.888-1.079 | 0.665 |  |  |  |
| Coaptation gap | 0.982 | 0.595-1.622 | 0.945 |  |  |  |
| Calcification of target area | 0.898 | 0.418-1.930 | 0.784 |  |  |  |
| G2 device use | 0.980 | 0.742-1.295 | 0.889 |  |  |  |
| Long clip use | 1.043 | 0.685-1.587 | 0.846 |  |  |  |
| Wide clip use | 0.930 | 0.667-1.298 | 0.671 |  |  |  |
| Number of clips | 1.468 | 1.095-1.968 | 0.010 | 1.213 | 0.864-1.703 | 0.265 |
| Atrial FMR cohort (n=300) |  |  |  |  |  |  |
| BMI 18.5 or higher | 0.884 | 0.457-1.711 | 0.715 |  |  |  |
| BNP median or higher | 1.193 | 0.593-2.400 | 0.620 |  |  |  |
| NT-Pro BNP median or higher | 1.198 | 0.597-2.406 | 0.611 |  |  |  |
| LVEF | 1.006 | 0.597-2.400 | 0.620 |  |  |  |
| LVEDV | 1.009 | 1.000-1.019 | 0.054 |  |  |  |
| LAVI | 1.003 | 0.999-1.006 | 0.104 |  | 0.996-1.009 | 0.393 |
| MV mean PG | 1.000 | 1.000-1.000 | 0.561 |  |  |  |
| TRPG | 1.010 | 0.988-1.032 | 0.372 |  | 0.989-1.023 | 0.516 |
| Stroke volume | 0.980 | 0.962-0.998 | 0.026 |  | 0.972-1.003 | 0.124 |
| EROA | 3.379 | 1.394-8.188 | 0.007 |  | 0.033-1.616 | 0.140 |
| MV orifice area | 1.099 | 0.936-1.292 | 0.249 |  |  |  |
| TAPSE | 0.988 | 0.929-1.050 | 0.695 |  |  |  |
| E wave | 1.008 | 0.999-1.016 | 0.085 |  |  |  |
| Septal E/e’ | 0.982 | 0.944-1.022 | 0.380 |  |  |  |
| Grasping length of AML | 0.945 | 0.881-1.014 | 0.116 |  |  |  |
| Grasping length of PML | 0.941 | 0.856-1.034 | 0.204 |  |  |  |
| Tenting height | 0.886 | 0.777-1.010 | 0.070 |  | 1.049-1.277 | 0.003 |
| Coaptation length | 0.880 | 0.681-1.136 | 0.326 |  |  |  |
| Coaptation gap | 1.103 | 0.511-2.378 | 0.803 |  |  |  |
| Calcification of target area | 0.319 | 0.040-2.564 | 0.283 |  |  |  |
| G2 device use | 1.004 | 0.595-1.694 | 0.989 |  |  |  |
| Long clip use | 1.691 | 0.761-3.757 | 0.197 |  |  |  |
| Wide clip use | 0.842 | 0.463-1.532 | 0.573 |  |  |  |
| Number of clips | 0.980 | 0.543-1.769 | 0.946 |  |  |  |

Abbreviations as seen in Table 1 and Table 2
